# Health-related behaviours of people with diabetes and those with cardiometabolic risk factors: results from SHIELD

**DOI:** 10.1111/j.1742-1241.2007.01588.x

**Published:** 2007-11

**Authors:** A. J. Green, D. D. Bazata, K. M. Fox, S. Grandy, for the SHIELD Study Group

**Affiliations:** 1 Midwestern Endocrinology, Overland Park KS, USA; 2 St Luke's South Primary Care, Overland Park KS, USA; 3 Strategic Healthcare Solutions, LLC Monkton, MD, USA; 4 Health Economics & Outcomes Research, AstraZeneca LP Wilmington, DE, USA

## Abstract

**Objectives:**

The study assessed knowledge, attitudes and behaviours towards health, diabetes, diet and exercise among respondents with type 2 diabetes mellitus and those with cardiometabolic risk factors.

**Methods:**

Respondents in the SHIELD study reported their health conditions, exercise, diet and weight loss. Three groups were assessed: (i) type 2 diabetes, (ii) high risk (HR) defined as 3–5 of the following factors: abdominal obesity, BMI ≥ 28 kg/m^2^, reported diagnosis of dyslipidaemia, hypertension, coronary heart disease or stroke and (iii) low risk (LR) defined as ≤ 2 factors. Comparisons across groups were made using analysis of variance.

**Results:**

More type 2 diabetes and HR respondents (> 46%) received recommendations to change their lifestyle habits (increase exercise and change eating habits), compared with < 29% of LR respondents, p < 0.0001. Less than 25% of respondents agreed that type 2 diabetes is not as serious as type 1 diabetes and > 85% agreed that obesity can aggravate or contribute to onset of chronic conditions. Mean number of healthcare visits was highest in type 2 diabetes (11.0) than HR (9.4) and LR (6.1) groups, p < 0.05. Type 2 diabetes and HR respondents were least likely to report exercising regularly (26%), compared with LR (37%), p < 0.05. More type 2 diabetes (70%) and HR (72%) respondents reported trying to lose weight vs. LR respondents (55%), p < 0.05.

**Conclusions:**

Type 2 diabetes and HR respondents reported attitudes and knowledge conducive to good health, but the majority of respondents did not translate these positive traits into healthy behaviour with respect to diet, exercise and weight loss.

**What's known**

Previous studies have demonstrated the benefits of changes in diet and physical activity on the prevention or delay of diabetes and its complications. Yet, the prevalence of diabetes and obesity are increasing globally.

**What's new**

This study provides evidence in a large, representative sample of individuals with type 2 diabetes or cardiometabolic risk factors of the current patterns of diet, exercise and other health-related behaviours to understand how a gap exists in managing diabetes and risk factors. This study demonstrates that there is a gap for most individuals as they report healthy attitudes and the health knowledge to manage their diabetes but have not translated this information into healthy behaviours.

## Introduction

Several studies have demonstrated the benefits of changes in diet and physical activity on the prevention or delay of diabetes and its complications (1–4). Lifestyle modification programmes with weight loss or exercise goals have shown a reduction in the incidence of diabetes in persons at high risk (HR) (2–3). This is important to public health as the number of Americans diagnosed with diabetes more than doubled from 1980 to 2002 and is projected to affect 300 million people globally by 2025 (5–6). Moreover, an estimated 41 million US adults aged 40–74 have prediabetes (5). This increasing prevalence of type 2 diabetes cannot be divorced from the rising prevalence of obesity and physical inactivity. Obesity and physical inactivity result in insulin resistance, which is one of the proximate underlying causes of type 2 diabetes (7–8). Moreover, an estimated 97 million US adults are overweight or obese (9), and approximately 75% of adult Americans have minimal physical activity (10). Thus, it is conceivable that modification of health attitudes and behaviour can mitigate both the individual and population risk for diabetes.

Little is known about the current patterns of diet, exercise and other health-related behaviours among the US population currently diagnosed with diabetes and those at risk for developing diabetes. It is also unclear as to whether those with greater knowledge or a positive attitude engage in healthier behaviours than those with limited knowledge or negative attitudes. It is important for clinicians, policy-makers and the public to know whether health behaviours are being implemented to delay development of diabetes among those with multiple cardiometabolic risk factors. The Study to Help Improve Early evaluation and management of risk factors Leading to Diabetes (SHIELD) provides a unique opportunity to characterise health-related behaviours in adults with type 2 diabetes mellitus and those at risk for type 2 diabetes, and to better understand where opportunities may exist for affecting behaviour change that can improve health outcomes.

The primary objective of this study was to report the knowledge, attitudes, and behaviours of survey respondents with diabetes and those at risk for a diagnosis of diabetes based on self-reported cardiometabolic risk factors. Additionally, the relationship between respondents’ health behaviour (e.g. diet, exercise) and knowledge regarding obesity and weight loss was evaluated.

## Research design and methods

### SHIELD survey

A detailed questionnaire (baseline survey) was mailed in 2004 to 22,001 individuals, age 18 years and older who were identified with diabetes or risk factors related to diabetes. The baseline survey assessed comorbid conditions, health status, knowledge, attitudes and current behaviours related to general health and diabetes, and exercise, diet and weight loss. Eighty per cent of baseline questionnaires were completed and included in the dataset. A detailed description of the SHIELD methodology has been published (11).

The sample source for the baseline survey was selected from respondents to a screening survey mailed to a stratified random sample of 200,000 US households, representative of the US population for age of head of household, income, household size, urban density and census region, identified by the Taylor Nelson Sofres National Family Opinion panel (TNS, Greenwich, CT). The screening questionnaire was completed by the head of household, who answered for up to four adult household members.

Respondents were classified according to diagnosis of diabetes (type 1 or type 2) and risk factors associated with increased risk of type 2 diabetes. Recognised risk factors, derived from the literature, national guidelines and expert opinion (12,13), included: (1) abdominal obesity (defined as waist circumference ≥ 97 cm for men and ≥ 89 cm for women), (2) BMI ≥ 28 kg/m^2^, (3) diagnosis of dyslipidaemia (cholesterol problems), (4) diagnosis of hypertension (high blood pressure) and (5) diagnosis of cardiovascular disease (defined as one or more of heart disease, myocardial infarction, narrow or blocked arteries, stroke, coronary artery bypass graft surgery, angioplasty, stents or surgery to clear arteries). Stepwise logistic regression analyses verified that these five risk factors were independently and equally predictive of diabetes diagnosis. Respondents with 0, 1 or 2 of the five risk factors were further classified as low risk (LR) for presence of diabetes and respondents with 3–5 risk factors were classified as HR.

### Statistical analyses

Three respondent groups were included in this analysis: type 2 diabetes (*n* = 3867), HR (*n* = 5419) and LR (*n* = 5683) groups. Comparisons across groups were conducted using analysis of variance with *post hoc* SNK Tukey multiple comparisons test. When the analysis of variance test indicated a statistical difference among the three groups, *ad hoc* statistical testing was done using chi-squared tests for categorical variables and *t*-tests for continuous variables to determine whether type 2 diabetes respondents were different from HR and LR respondents separately. Statistical significance was set *a priori* as p < 0.05.

## Results

In all, 3867, 5419 and 5683 type 2 diabetes, HR and LR respondents, respectively, completed the baseline questions on health knowledge, attitudes and behaviours. The majority of respondents were women and white. Type 2 diabetes respondents were more likely to have a lower household income and less education than HR or LR respondents, p < 0.01 (Table 1).

**Table 1:** Characteristics of SHIELD type 2 diabetes mellitus, high-risk and low-risk respondents

Characteristics	Type 2 diabetes (*n* = 3867)	High risk (*n* = 5419)	Low risk (*n* = 5683)
Age, years, mean (SD)	60.2 (13.1)	59.0 (14.7)*	47.1 (16.4)**
Men (%)	42	43	35**
Whites (%)	85	88*	88**
Education, % with at least some college	64	68*	74**
Income, % with household income ≥ $40,000/year	47	54*	64**
Geographic region (%)			
Northeast (New England, Middle Atlantic)	20	20	19
North Central	24	25	25
South Central	17	17	16

* p < 0.05 for comparison of type 2 diabetes to high risk;

** p < 0.05 for comparison of type 2 diabetes to low risk.

### Attitudes about diabetes

Approximately 22% of type 2 diabetes respondents agreed (somewhat or strongly) that type 2 diabetes is not as serious as type 1 diabetes compared with 11.2% of HR and 10.8% of LR respondents (p < 0.05, Table 2). About 10% of respondents with or without diabetes agreed that diabetes is only a sugar disease. Most respondents in each group (> 85%) agreed that obesity can aggravate or contribute to the onset of chronic diseases.

**Table 2:** Knowledge, attitudes and behaviours for type 2 diabetes, high-risk and low-risk respondents in the SHIELD survey*

	Type 2 diabetes	High risk	Low risk	ANOVA, p-value
**Attitudes**				
Type 2 is not as serious as type 1 diabetes†	22.2	11.2	10.8	0.0001
Diabetes is only a sugar disease†	9.7	10.1	10.0	0.81
Obesity can aggravate or contribute to onset of chronic diseases†	87.1	86.4	88.7	0.001
Compared with 12 months ago, health now is:				
Much or somewhat better	26.3	24.0	21.9	0.0001
About the same	48.2	52.7	64.9	
Much or somewhat worse	25.5	23.4	13.2	
Compared with now, expect health in 12 months to be:				
Much or somewhat better	31.4	31.3	30.3	0.0001
About the same	55.1	56.5	63.9	
Much or somewhat worse	13.5	12.2	5.8	
**Knowledge**				
Healthcare professional recommended increase in exercise in the past 12 months‡	62.5	56.1	28.3	0.0001
Healthcare professional recommended change in eating habits during the last 12 months‡	56.3	46.9	18.4	0.0001
**Behaviours**				
Exercise				
Regularly, > 6 months	18.9	18.7	29.1	0.0001
Regularly, < 6 months	7.5	8.1	8.0	
Some, but not regularly	44.3	42.0	39.2	
Currently do not	29.2	31.3	23.7	
Physical activity during the last 7 days				
Active‡	12.7	14.9	24.1	0.0001
Inactive‡	87.3	85.1	75.9	
Try to make healthy food choices§	78.1	70.1	68.3	0.0001
Follow a prescribed eating plan§	32.6	13.5	7.1	0.0001
Weight loss				
Tried to lose weight in the past 12 months	69.6	71.5	54.7	0.0001
Tried to keep from gaining weight in last 12 months	74.9	74.0	61.7	0.0001
Maintained desired weight for > 6 months	33.7	27.0	49.9	0.0001
If not, due to what you eat	83.9	86.7	80.6	0.0001
If not, due to a hormone or metabolism problem	28.3	26.1	23.3	0.001
Seriously considering reaching weight goal in the next 6 months	57.3	61.0	45.9	0.0001

* Sample size vary slightly for each survey question;

† Agree somewhat or strongly;

‡ Yes;

§ Most of the time or always.

### Attitudes about health

Most respondents reported that their health had remained about the same compared with 12 months ago; however, significantly fewer type 2 diabetes respondents (48%) than HR (53%) and LR (65%) respondents reported this, p < 0.0001 (Table 2). Similarly, the majority of respondents stated that they expect their health to be about the same next year. Yet, significantly more respondents in the type 2 diabetes and HR groups reported that their health would be worse next year than now compared with the LR group (13.5% and 12.2%, respectively, vs. 5.8%, p < 0.05).

### Exercise: knowledge and behaviour

More type 2 diabetes (62%) and HR (56%) respondents reported receiving a recommendation by a healthcare professional to increase the amount they exercise, compared with LR respondents (28%) (p < 0.05, Table 2). However, actual behaviour towards exercise showed the opposite pattern. Type 2 diabetes and HR respondents were the least likely to report exercising regularly for at least the past 6 months (26.4% and 26.8% respectively), compared with LR respondents (37.1%, p < 0.0001 for both). Moreover, fewer type 2 diabetes (12.7%) and HR (14.9%) respondents reported doing moderate or vigorous physical activities in the past 7 days than LR respondents (24.1%, p < 0.05) (Table 2).

### Diet: knowledge and behaviour

More type 2 diabetes respondents (56.3%) than HR (46.9%) or LR (18.4%) respondents reported that in the past 12 months, a healthcare professional recommended that they change what they eat or reduce the amount they eat (p < 0.05, Table 2). A majority of type 2 diabetes respondents reported healthy behaviours regarding diet: 78% reported trying to make healthy food choices most of the time or always. Fewer HR and LR respondents reported these healthy diet behaviours compared with type 2 diabetes respondents (p < 0.05); 70.1% and 68.3%, respectively, reported trying to make healthy food choices (Table 2). Only 32.6% of type 2 diabetes, 13.5% of HR and 7.1% of LR groups reported following an eating plan most of the time or always that was prescribed by a physician, p < 0.05.

### Weight loss behaviour

More respondents in the type 2 diabetes (69.6%) and HR groups (71.5%) reported trying to lose weight in the past 12 months, compared with respondents in the LR group (54.7%) (p < 0.0001, Table 2). Likewise, 74.9% of type 2 diabetes and 74.0% of HR respondents were trying to keep from gaining weight, compared with 61.7% of LR respondents, p < 0.0001 (Table 2). Most respondents in each group who had not been able to maintain their desired weight indicated that it was because of what they ate, whereas approximately one-quarter attributed it to a hormone or metabolism problem.

### Healthcare-seeking behaviour

**Figure 1:**
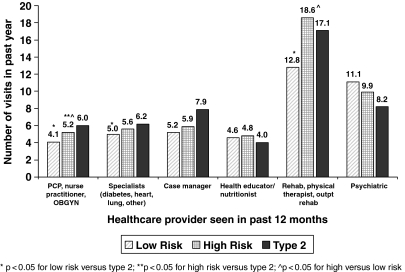
Mean number of provider visits for type 2 diabetes, high-risk and low-risk groups

Among respondents who had at least one visit to a healthcare provider (i.e. primary care physician, nurse practitioner, case manager, health educator, nutritionist/dietician, physical therapist, endocrinologist, cardiologist, pulmonologist, psychiatrist/psychologist, OB/GYN or other specialist) during the previous 12 months, the mean total number of healthcare visits was highest for respondents with type 2 diabetes (11.0), followed by those in the HR (9.4) and LR groups (6.1) (p < 0.05). Respondents in all groups had more visits to providers for rehabilitation and physical therapy than for any other type of provider (Figure 1). The providers with the least number of visits were diabetes educators, nutritionists and dieticians with 4–5 visits in the past 12 months for each group (Figure 1). Type 2 diabetes respondents had an average of six visits to primary care providers and six visits to specialists in the past 12 months, compared with 5.2 and 4.1 primary care visits and 5.6 and 5.0 specialists visits for HR and LR, respectively, p < 0.001 for HR vs. type 2 and p < 0.0001 for LR vs. type 2.

### Subgroup analysis: linking attitude to behaviour in type 2 diabetes

The association between type 2 diabetes respondents’ attitudes about weight loss and obesity and actual exercise and diet behaviour was assessed by stratifying their responses (agree vs. disagreed/neutral) to two survey items: ‘obesity aggravates or contributes to the onset of chronic diseases’ and ‘the inability to keep weight off is due to a hormone or metabolism problem’.

Respondents with type 2 diabetes indicated that they understood the impact of obesity on health with 87.1% responding that they agree that obesity aggravates or contributes to the onset of chronic conditions. Those who agreed also reported better exercise and eating habits, than those who disagreed, p < 0.05 (Table 3). More respondents who agreed also reported trying to lose weight (70.5%) or keep from gaining weight (76.3%) in the previous 12 months than those who disagreed (64.6% and 66.3%, respectively, p < 0.05). However, when asked about ability to maintain weight, a similar percentage of type 2 diabetes respondents who agreed or disagreed had not maintained their desired weight for a period of 6 months or longer (agree, 66.3%; disagree, 66.7%, p = 0.88) and a similar percentage of respondents agreeing or disagreeing ascribed the inability to maintain desired weight to what they eat or their level of exercise.

**Table 3:** Relationship between obesity knowledge and diet and exercise behaviours among type 2 diabetes respondents

	Obesity aggravates/ contributes to onset of chronic conditions		Inability to keep weight off due to hormone or metabolism problem	
		
	Agree (*n* = 3317)*	Disagree or neither (*n* = 483)*	Agree (*n* = 1005)*	Disagree or neither (*n* = 2782)*
**Exercise**				
Healthcare provider recommended increase in exercise (yes)	63.8**	55.3	68.1**	60.6
Exercise routine				
Some, not regularly	44.1**	46.5	46.0**	43.9
Regularly, > 6 months	19.7	13.0	16.0	20.0
Regularly, < 6 months	7.8	6.1	7.7	7.5
No, thinking of starting within next 6 months	15.8	16.7	18.8	14.8
No, do not intend to start within next 6 months	12.6	17.7	11.5	13.9
**Diet**				
Healthcare provider recommended changes in diet (yes)	57.4**	49.4	62.0**	54.3
Using a prescribed eating plan? (not currently)	48.0**	56.1	48.1	49.4
Try to make healthy food choices				
Always or most of the time	79.1**	72.0	76.5	78.6
Sometimes or not currently	20.9	28.0	23.5	21.4
**Weight loss**				
Tried to lose weight during past 12 months	70.5**	64.6	76.4**	67.2
Tried to keep from gaining in past 12 months	76.3**	66.3	78.8**	73.7
Have maintained desired weight > 6 months? (no)	66.3	66.7	73.1**	64.0
If no, is it due to:				
What you eat and level of exercise? (yes)	85.2**	76.1	83.3	84.2
Undiagnosed hormone or metabolic problem? (yes)	27.3**	35.8	48.8**	19.7

* *n* varies slightly from question to question;

** p-value < 0.05 for agree vs. disagree/neutral.

Values are given in percentage.

For type 2 diabetes respondents, responses to the survey item: ‘the inability to keep weight off is due to a hormone or metabolism problem’ were assessed to determine if the perception of an external, uncontrollable force is associated with diet and exercise behaviour (Table 3). Only 38.0% of those with type 2 diabetes disagreed with this statement, whereas 62.0% either agreed or were neutral, indicating that most respondents’ attitude was to associate weight problems with physiological conditions.

A greater percentage of patients who agreed that their inability to lose weight was due to a metabolic or hormone problem reported trying to lose weight (76.4%) or keep from gaining it (78.8%) than those who disagreed (67.2% and 73.7% respectively), p < 0.05 (Table 3). In addition, more type 2 diabetes respondents who agreed had been able to maintain a desired weight for a period of 6 months or longer (73.1%), compared with those who disagreed (64.0%), p < 0.05. Approximately half of the respondents who agreed to the statement and who had not been able to maintain a desired weight believed that this was due to an undiagnosed hormone or metabolism problem.

## Discussion

Data from the SHIELD survey demonstrated that knowledge, attitudes and behaviours are similar among the type 2 diabetes and HR groups and vary considerably from those in the LR group. The similarities between the type 2 diabetes and HR groups were remarkable. These respondents were aware of the behaviours necessary to maintain or improve their health but were not reporting behaviours in alignment with their knowledge or attitudes. Approximately one-quarter of type 2 diabetes and HR respondents reported that their health had worsened in the last 12 months, and they were the least likely to report regular exercise in the past 12 months and the most likely to state that they did not intend to start exercising. On the other hand, about one-half of these respondents reported having been advised to change their diet and exercise practices by healthcare professionals. The majority of these respondents reported trying to make healthy choices and lose weight, but most have not been successful with maintaining desired weight or exercising regularly.

As diabetes is a self-managed disease in which day-to-day decisions affecting health and well-being are made by patients themselves (14), it is important to highlight, and perhaps alarming, that a high proportion of individuals who reported a clear understanding of healthy attitudes and had been advised by healthcare professionals did not report healthy behaviours. Many of these behaviours involve routine daily activities, such as diet and exercise, which the SHIELD data showed were not embraced even by those who understood the negative impact of obesity on chronic conditions. The majority of respondents with type 2 diabetes agreed that obesity aggravates or contributes to chronic disease, but their behaviours associated with diet and exercise were not consistent with that attitude. Most telling was that respondents who disagreed that obesity aggravates or contributes to chronic conditions reported similar diet and exercise behaviours as those who agreed, showing that this attitude alone does not result in a demonstrable improvement in self-reported health behaviour. Moreover, the uncertainty or belief of most respondents with type 2 diabetes that a physiological condition beyond their control (hormone or metabolism problem) contributes to an inability to lose weight would be considered a likely impediment to behaviour change.

The present findings are consistent with other studies concluding that knowledge alone does not necessarily alter behaviour or predict outcomes (15–17). Heisler et al. (17) demonstrated that knowledge of one's HbA_1c_ level alone was insufficient to translate increased understanding of diabetes care into the increased confidence and motivation necessary to improve patients’ diabetes self-management. Identifying the knowledge and attitudes that do lead to behaviour change is clearly difficult. The SHIELD study demonstrated that knowledge and attitudes about the impact of obesity on chronic disease as well as being advised by healthcare professionals to increase exercise and change eating patterns was not linked to behaviour change. Future investigations may need to include items on goals related to appearance and taking care of one's health to determine if these factors are associated with health-enhancing behaviours.

While a minimum knowledge threshold is needed to achieve long-term healthy behaviour patterns (15,18), addressing patients’ own perceptions of barriers, as well as their values, motivations and goals, has been found to be better than knowledge alone in changing behaviour (17). Estabrooks et al. (19) found that when patients selected personally appropriate goals, they achieved significant behaviour change. Anderson and Funnell (14) suggested that effective diabetes care involve an empowerment approach that requires patients and healthcare professionals to collaborate in the development of self-management plans that integrate the clinical expertise of healthcare professionals with the concerns, priorities and resources of the patient.

More effective interventions to improve health-related behaviours are clearly needed, both for individuals with diabetes and those at HR. It is important to note that SHIELD HR respondents were very similar to the type 2 diabetes respondents in their knowledge, attitudes and behaviours, such that HR respondents exhibited a similar gap in attitudes translating to healthy behaviours which may increase their risk of developing diabetes. The SHIELD data also show that the average number of visits to nutritionists, health educators or case managers was low relative to other healthcare providers. In the context of the exercise and diet findings, we suggest that greater utilisation of nutritional and health education may result in improved implementation of healthy behaviours.

## Conclusion

Type 2 diabetes respondents and those at-risk for diabetes largely reported healthy attitudes and knowledge towards health and diabetes but these positive traits did not translate into healthy behaviours in diet, exercise and weight loss. The present findings highlight the need for the development of more effective interventions and strategies for diabetes prevention and treatment by closing the gap between knowledge and attitudes vs. healthy behaviours.
